# Lipid metabolism mediating oxaliplatin resistance in colorectal cancer

**DOI:** 10.3389/fonc.2026.1976409

**Published:** 2026-10-22

**Authors:** Siqi Han, Liqiang Gu, Ziyu Ren, Jingxian Hu, Ying Zhao, Zhenying Zhao, Yijia Wang

**Affiliations:** 1 Graduate School of Tianjin University of Traditional Chinese Medicine, Tianjin, China; 2 Department of Colorectal Surgery, Tianjin Union Medical Center, The First Affiliated Hospital of Nankai University, Tianjin, China; 3 Department of Pharmacy, Tianjin Union Medical Center, The First Affiliated Hospital of Nankai University, Tianjin, China; 4 Department of Pathology, Tianjin Union Medical Center, The First Affiliated Hospital of Nankai University, Tianjin, China

**Keywords:** colorectal cancer, fatty acid oxidation, lipid metabolism, oxaliplatin, reactive oxygen species

## Abstract

**Introduction:**

As a core drug in chemotherapy regimens for colorectal cancer, the clinical efficacy of oxaliplatin is compromised by multiple resistance mechanisms. Lipid metabolism, a crucial component of metabolic pathways, is known to affect the efficacy of various chemotherapeutic agents. However, to the best of our knowledge, the role of lipid metabolism in oxaliplatin resistance remains unreviewed.

**Areas covered:**

In this review, we analyze the relationship between lipid metabolism and oxaliplatin efficacy from the perspectives of DNA damage, ferroptosis, ROS accumulation, cellular dormancy, antitumor immunity, vascular remodeling, and autophagy. This review covers nearly all known oxaliplatin resistance factors, summarizing the regulatory role of lipid metabolism across both basic and clinical studies. Given that clinical studies remain relatively scarce compared to basic research, the impact of factors directly regulated by lipid metabolism on clinical outcomes is analyzed. Based on this, their relationship with oxaliplatin efficacy is inferred, and potential directions for future clinical research are highlighted.

**Expert opinion/commentary:**

By summarizing the relationship between lipid metabolism and oxaliplatin resistance, this review provides an important theoretical basis for improving chemotherapy efficacy in colorectal cancer and developing novel combination therapies based on metabolic interventions.

## 1 Introduction

Oxaliplatin (OXA) is a core drug in chemotherapy regimens for colorectal cancer (CRC). When combined with a fluoropyrimidine, it forms the basis of the FOLFOX and CAPOX regimens, which are widely used as postoperative adjuvant therapy for patients with stage III and selected high-risk stage II CRC, as well as for the systemic treatment of unresectable or recurrent metastatic disease (1). However, the therapeutic goals of OXA treatment and the clinical manifestations of resistance differ between these two settings. In patients whose primary tumor has been completely resected, with no metastases detected by imaging but pathological findings indicating a risk of recurrence, adjuvant chemotherapy aims to eradicate residual disease that is not visible on conventional imaging, including micrometastases, circulating tumor cells, and molecular residual disease (MRD). By contrast, in patients with unresectable metastatic lesions, treatment is primarily intended to shrink or stabilize existing tumors, delay disease progression, and make surgical resection possible for selected patients. Evidence from studies such as DYNAMIC (2) and GALAXY (3) indicates that postoperative circulating tumor DNA (ctDNA) positivity is strongly associated with an increased risk of recurrence, demonstrating that the absence of radiologically detectable disease does not necessarily mean that no residual tumor cells remain in the body. Accordingly, the failure of metastatic lesions to shrink after an initial course of treatment is more likely to reflect primary resistance, whereas recurrence several years after surgery may involve residual tumor cells that survive chemotherapy, remain dormant for an extended period, and subsequently reactivate (4).

Platinum-based chemotherapeutics can upregulate ROS via multiple molecular pathways. These mechanisms are also operative for OXA triggered cytotoxicity. For instance, OXA is capable of forming conjugates with glutathione (GSH), which results in massive intracellular depletion of this thiol peptide and consequently elevates intracellular ROS levels by impairing cellular antioxidant capacity (5). However, tumor cells can evade OXA induced cytotoxicity through reduced intracellular drug exposure, enhanced DNA damage repair and antioxidant capacity, suppression of apoptosis and ferroptosis, activation of cytoprotective autophagy, and maintenance of cancer stemness. Treatment pressure may also select for slowly proliferating drug tolerant persister (DTP) cells or cells with a dormancy like phenotype. These cells temporarily exit active cell cycling and rely on metabolic adaptation, autophagy, and support from the tumor microenvironment to survive; once treatment is discontinued, they may resume proliferation. For circulating or disseminated tumor cells that have detached from the primary tumor, resistance to anoikis is also an important condition for the establishment of occult metastases. For example, CPT1A mediated fatty acid oxidation (FAO) can suppress anoikis in CRC cells and thereby promote metastatic dissemination (6). At the same time, poor perfusion and hypoxia caused by abnormal tumor vasculature (7), immunosuppressive myeloid cells (8), and metabolic substrates supplied by stromal cells and adipocytes may collectively limit the therapeutic efficacy of OXA (9).

These diverse resistance phenotypes are not mutually exclusive, and metabolic reprogramming represents a shared molecular foundation underlying multiple resistance‑related behaviors. Among various metabolic perturbations, lipid metabolism is of particular interest: lipids contribute to membrane biogenesis, energy production, signal transduction, and redox homeostasis, and may therefore modulate tumor responsiveness to OXA at three critical steps: initiation of drug inflicted damage, damage repair, and the execution of cell death (10). Clinical evidence indicates that, among patients with stage III or high risk stage II CRC, higher preoperative levels of triglycerides and apolipoprotein B were associated with shorter disease free survival (DFS) and overall survival (OS). This clinical association is prognostic and does not establish OXA-specific resistance (11). Similarly, in a cohort receiving adjuvant FOLFOX chemotherapy, baseline body mass index (BMI) was not significantly associated with overall DFS, whereas marked weight gain during treatment was associated with poorer DFS among patients who were overweight or obese (12). These observations suggest a relationship between obesity, dyslipidemia, and chemotherapy outcomes. Its interpretation should account for tumor stage, fat distribution, nutritional status, and treatment regimen; obesity alone cannot be considered evidence of OXA resistance. By contrast, adipocyte derived exosomal microsomal triglyceride transfer protein (MTTP) promotes OXA resistance by strengthening the xCT/GPX4 dependent antiferroptotic defense system while reducing ACSL4 expression and lipid peroxidation. This mechanism provides more direct translational evidence linking host adiposity, tumor lipid metabolism, and the response to OXA (9). Lipid metabolic reprogramming in CRC involves multiple interconnected processes, including fatty acid synthesis and uptake, triglyceride synthesis and lipid droplet storage, FAO, and membrane phospholipid peroxidation.

To delineate how lipid metabolism drives OXA resistance, this review focuses on several core molecular modules: lipid droplet homeostasis regulated by LPIN1/DGAT2, GPAT3, and LPCAT2; FAO mediated by CPT1/CPT2; and the ferroptosis threshold jointly determined by ACSL4/LPCAT3 and the SLC7A11–GSH–GPX4 axis. These three functional modules correspond to distinct biological processes. First, LPIN1/DGAT2, GPAT3 and LPCAT2 constitute the core machinery governing lipid‑droplet biogenesis and turnover: LPIN1 and DGAT2 sequentially promote triacylglycerol synthesis for lipid‑droplet storage, GPAT3 initiates glycerolipid biosynthesis, whereas LPCAT2 supports phosphatidylcholine production linked to lipid‑droplet membranes. Together they shape fatty acid sequestration, lipotoxicity buffering and immunogenic cell death. Second, CPT1/CPT2‑driven FAO facilitates mitochondrial fatty‑acid catabolism, sustaining NADPH‑dependent redox homeostasis and DNA‑repair‑related acetyl‑CoA supply under chemotherapeutic stress. Third, the ACSL4/LPCAT3 and SLC7A11–GSH–GPX4 axis jointly sets the ferroptosis threshold: ACSL4‑LPCAT3 generates peroxidizable PUFA‑PL substrates, while the SLC7A11‑GSH‑GPX4 system executes lipid‑peroxide detoxification. The balance between substrate supply and antioxidant capacity ultimately dictates whether OXA‑challenged CRC cells undergo ferroptosis.

Through distinct but interconnected mechanisms, these pathways can influence OXA induced ROS accumulation, immunogenic cell death, the survival of residual tumor cells, and the execution of ferroptosis. Their activities may also vary according to the molecular background of the tumor, the surrounding microenvironment, and the stage of treatment. For example, OXA can induce CPT1B/CPT2 associated fatty acid catabolism, which buffers ROS accumulation by maintaining NADPH availability and redox homeostasis. Inhibition of this adaptive response has been shown to enhance the antitumor activity of OXA in preclinical models (13).

Rather than acting merely as an auxiliary pathway in OXA resistance, lipid metabolism participates broadly in DNA damage repair, ROS clearance, ferroptosis escape, cytoprotective autophagy, reduced tumor immunogenicity, vascular and hypoxic remodeling, and the dormancy and subsequent recurrence of residual tumor cells. Accordingly, this review examines how alterations in lipid metabolism affect OXA induced cell death and tumor microenvironmental responses. It further distinguishes among pre-existing metabolic states before treatment, reversible adaptations induced by therapeutic pressure, and stable acquired resistance, in order to evaluate the evidence and limitations of these factors as biomarkers of therapeutic efficacy and targets for combination therapy.

The principal lipid-metabolic determinants discussed in this review, their functions, and the strength and limitations of the evidence linking them to OXA response are summarized in **Table 1**.

**Table 1:** Lipid metabolic factors and pathways implicated in oxaliplatin response and resistance in colorectal cancer.

Molecule or axis	Principal lipid-metabolic function	Relationship to OXA response	Evidence tier, basis, and limitations
p53-ABCA1-SREBP2	ABCA1 promotes retrograde cholesterol transport to the ER; this restrains SREBP2 maturation and mevalonate/cholesterol biosynthesis.	Loss of wild-type p53 activity may release this biosynthetic brake and facilitate membrane renewal and stress adaptation; a direct effect on OXA resistance has not been demonstrated.	Mechanistic cancer evidence, but not OXA-specific; selected mutant-p53/mevalonate findings derive from non-CRC models.
CPT1B/CPT2	CPT1B forms long chain acylcarnitines at the outer mitochondrial membrane; CPT2 regenerates acyl-CoA in the matrix for beta oxidation.	OXA-induced FAO supports bioenergetic and NADPH/GSH homeostasis, limits ROS accumulation and apoptosis, and reduces OXA efficacy; pharmacological or genetic inhibition sensitizes tumors.	Direct preclinical gastrointestinal-cancer evidence, including xenograft and patient derived models; no prospective CRC trial validation.
CPT2 downregulation	Reduces mitochondrial long-chain FAO capacity and produces a chronic oxidative/metabolic state distinct from acute OXA-induced FAO activation.	Chronic ROS can activate Wnt/beta-catenin-driven glycolysis and stemness; CPT2 restoration increases OXA sensitivity.	Direct CRC preclinical evidence with clinical association; direction is context- and time-dependent and should not be conflated with acute CPT1B/CPT2 induction.
CPT1A	Controls mitochondrial entry of long chain fatty acids through acylcarnitine formation.	CPT1A-dependent FAO suppresses anoikis and supports metastatic dissemination, but its causal role in OXA resistance was not tested.	CRC metastasis evidence; contextual rather than OXA specific.
GPAT3	Acylates glycerol-3-phosphate to form lysophosphatidic acid, initiating glycerolipid synthesis and promoting triacylglycerol/lipid-droplet formation.	Acetylation-dependent GPAT3 activity increases lipid droplets, reduces apoptosis and calreticulin exposure, weakens antitumor immunity, and promotes chemoresistance.	Direct CRC chemotherapy evidence in cell and animal models; lipid droplet to FAO flux and GPAT3 specific ferroptosis escape remain unproven.
LPIN1/DGAT2	LPIN1 converts phosphatidic acid to diacylglycerol; DGAT2 acylates diacylglycerol to triacylglycerol for lipid-droplet storage.	This lipid-droplet program buffers lipotoxic and oxidative stress in therapy-resistant cells; disrupting it increases lipid ROS and ferroptotic vulnerability.	Therapy-resistant CRC/DTP evidence; not OXA exclusive and not yet clinically validated.
ELF1-ACSL5-lipid-droplet axis	ELF1 transcriptionally activates ACSL5. ACSL5 promotes the channeling of oxidized lipids into lipid droplets, supporting redox buffering.	In 5-FU/OXA induced dormant CRC cells, the axis lowers intracellular ROS and sustains dormancy and residual survival; disrupting ACSL5 or lipid-droplet formation increases ROS.	Direct mechanistic evidence from PDX-derived CRC cells, LoVo cells, lipidomics and post-chemotherapy patient specimens; the treatment model used combined 5-FU/OXA and does not establish an OXA monotherapy-specific effect.
LPCAT2	Reacylates lysophosphatidylcholine to phosphatidylcholine and supports membrane remodeling and lipid-droplet biogenesis.	LPCAT2 dependent lipid droplets attenuate ER stress, caspase activation and calreticulin exposure, thereby reducing immunogenic cell death and supporting chemoresistance.	Direct CRC chemotherapy evidence; the study addresses a broader chemotherapy response rather than a universally OXA specific pathway.
ACSL4	Activates arachidonic, adrenic and other PUFAs to PUFA-CoA, supplying acyl chains for oxidizable membrane phospholipids.	CDK1-mediated ACSL4 degradation lowers PUFA-phospholipid abundance, lipid ROS and ferroptosis; restoring ACSL4 activity enhances OXA sensitivity.	Direct CRC OXA evidence in cellular and *in vivo* models; strong preclinical support without prospective clinical validation.
LPCAT3	Facilitates incorporation of PUFA acyl chains into phospholipids, expanding the membrane substrate pool for lipid peroxidation.	Higher LPCAT3 dependent PUFA-phospholipid availability is expected to lower the ferroptosis threshold when detoxification is insufficient.	Established ferroptosis mechanism, but LPCAT3 has not been independently validated as a causal OXA resistance determinant in CRC.
SLC7A11/SLC3A2-GSH-GPX4	System xc- imports cystine for GSH synthesis; GPX4 uses GSH to reduce phospholipid hydroperoxides.	Activation of this detoxification arm removes lipid peroxides, suppresses ferroptosis and increases OXA tolerance.	Canonical ferroptosis-defense mechanism; direct OXA CRC support is supplied by the upstream pathways summarized separately below.
KIF20A/NUAK1-NRF2-GPX4	Stress-response signaling reinforces NRF2-dependent antioxidant and GPX4 activity.	Suppression of this pathway induces ferroptosis and increases CRC sensitivity to OXA.	Direct OXA-CRC preclinical evidence; the relative contributions of SLC7A11, GPX4 and other NRF2 targets require separation.
F. nucleatum-E-cadherin/beta-catenin/GPX4	Microbial signaling stabilizes a cell adhesion/transcriptional program that increases GPX4-dependent lipid-peroxide detoxification.	F. nucleatum suppresses ferroptosis and promotes OXA resistance.	Direct OXA CRC experimental evidence; effects may depend on bacterial strain, colonization level and tumor context.
Exosomal MTTP-PRAP1-ZEB1	Adipocyte-derived exosomal MTTP transmits a host lipid-handling signal; MTTP canonically transfers neutral lipids during lipoprotein assembly.	The pathway increases xCT/GPX4 and decreases ACSL4, PUFA-phospholipids and lipid ROS, thereby suppressing ferroptosis and promoting OXA resistance.	Direct evidence from CRC cells, patient-derived organoids and obese-mouse models; prospective patient validation is lacking.
FAO-ACLY-acetyl-CoA-p300/H3K27ac-Nanog	FAO derived carbon supports acetyl-CoA production and histone acetylation at the Nanog promoter.	The pathway promotes p21/p27-associated G0/G1 dormancy and may support persistence, but it has not been demonstrated as an OXA-induced dormancy mechanism.	CRC serum deprivation model; contextual evidence, not OXA-specific.
IL33-mTOR/PPARgamma-DGAT1/2	Activated hepatic stellate cells induce neutrophil triacylglycerol/lipid-droplet synthesis and lipid transfer to dormant CRC cells.	Transferred lipids restore FAO and eicosanoid production and promote dormant-cell reactivation and metastatic regrowth.	CRC liver-metastasis evidence; relevant to recurrence biology but not directly tested during OXA treatment.
COX-2-PGE2-VEGF	Converts arachidonic-acid signaling into prostaglandin driven inflammation and angiogenic VEGF output.	Celecoxib combined with OXA produced greater tumor inhibition than either agent alone in an HT29 xenograft model.	Direct combination-treatment evidence is preclinical; contributions of PGE2 suppression, perfusion and tumor-cell-intrinsic effects were not fully separated.
HSF1/miR-135b-5p-MUL1-ULK1	Stabilizes ULK1 and activates cytoprotective autophagy; a lipid droplet/lipophagy connection remains hypothetical.	The pathway promotes OXA resistance by sustaining protective autophagy.	Direct OXA CRC evidence for autophagy; no direct proof that lipophagy-derived fatty acids feed FAO in this setting.
PLA2G16	Lipid-hydrolysis/remodeling enzyme; loss alters triacylglycerol, phosphatidylcholine and phosphatidylethanolamine profiles.	PLA2G16 deficiency increases OXA-induced growth inhibition and apoptosis and is accompanied by altered autophagy-related markers.	Direct CRC cell and xenograft evidence; the responsible lipid product and causal dependence on autophagy or lipophagy remain unresolved.

Evidence was classified as direct only when OXA was tested in CRC or gastrointestinal-cancer models. Contextual evidence was retained when it informs the biological interpretation but does not establish OXA-specific causality. The ELF1-ACSL5 evidence derives from combined 5-FU/OXA exposure and should not be interpreted as an OXA-monotherapy-specific mechanism. ACLY, ATP citrate lyase; CRC, colorectal cancer; DTP, drug-tolerant persister; ER, endoplasmic reticulum; FAO, fatty acid oxidation; GSH, glutathione; LD, lipid droplet; OXA, oxaliplatin; PUFA, polyunsaturated fatty acid; ROS, reactive oxygen species.

## 2 Lipid metabolic regulation of DNA damage induced apoptosis

In both clinical settings described above, whether the response of tumor cells is shaped by pre-existing metabolic states, reversible treatment-induced adaptations, or mechanisms of stable acquired resistance, the central cellular question remains the same: whether drug induced DNA damage can be effectively translated into irreversible cell death. After entering the cell, OXA forms platinum DNA adducts that interfere with DNA replication and transcription, stall replication forks, and may generate secondary DNA double strand breaks when stalled forks collapse. ATR primarily responds to replication stress and the single stranded DNA generated at stalled replication forks, whereas ATM is preferentially activated after double-strand breaks have formed. The ATR–Chk1 and ATM–Chk2 pathways subsequently establish cell cycle checkpoints and regulate downstream molecules such as p53, p21, and CDC25C. When the damage remains repairable, checkpoint-mediated cell cycle arrest provides additional time for the Fanconi anemia pathway, nucleotide excision repair, and homologous recombination (HR) to remove or repair the lesions. When damage persists or exceeds the cell’s repair capacity, p53 can induce PUMA, NOXA, and BAX, thereby promoting BAX/BAK mediated mitochondrial outer membrane permeabilization and apoptosis. Thus, following OXA induced DNA damage, whether a cell undergoes repair, prolonged cell cycle arrest, senescence, or apoptosis ultimately depends on the balance among the extent of DNA damage, the strength of checkpoint signaling, and the capacity for DNA repair.

Direct evidence from CRC models shows that OXA resistant cells exhibit increased CHK2 phosphorylation, PARP1 mediated poly(ADP-ribosyl)ation (PARylation), and HR activity. Inhibition of CHK2 reduces PARylation, FANCD2 monoubiquitination, and HR efficiency while partially restoring sensitivity to OXA. These findings indicate that sustained activation of the CHK2–PARP1–DNA repair axis enables resistant cells to survive platinum induced DNA damage (14). Thus, DNA damage caused by OXA does not inevitably lead to cell death. When cell cycle checkpoints and DNA repair programs remain persistently active, tumor cells may exploit cell cycle arrest to gain time for damage repair, preventing DNA lesions from culminating in cell death and thereby sustaining survival and promoting treatment tolerance.

The functional status of p53 can modify the response to OXA by coupling DNA damage signaling to lipid metabolic adaptation.p53 status also modifies the response to OXA: p53-proficient CRC cells may undergo p53–p21-dependent arrest or senescence (15), whereas p53-R273H promotes mitochondrial biogenesis, oxidative phosphorylation, and migration, indicating a variant-specific gain-of-function response (16).

More directly relevant to lipid biosynthesis, wild type p53 transcriptionally induces ATP-binding cassette transporter A1 (ABCA1), which promotes retrograde cholesterol transport from the plasma membrane to the endoplasmic reticulum and thereby suppresses sterol regulatory element-binding protein 2 (SREBP2) maturation. This constrains mevalonate pathway activity and the synthesis of cholesterol and isoprenoids (17). Conversely, selected mutant p53 proteins cooperate with SREBPs to activate mevalonate-pathway genes in breast cancer models, providing contextual rather than CRC-specific evidence (18). Loss or functional impairment of p53 may therefore release the wild-type metabolic brake, while specific mutant proteins may actively reinforce lipid biosynthesis. Either change could support cholesterol synthesis and membrane-lipid renewal during OXA exposure, thereby preserving membrane biogenesis, organelle function, and stress adaptation. Consistent with the relevance of lipid remodeling to OXA stress, lipidomic and transcriptomic analyses of OXA treated HT29 cells identified increased phospholipid classes and extensive reprogramming of lipid metabolic pathways (19). Because the HT29 study did not stratify TP53 status or test causality, the relationship between p53–SREBP/mevalonate signaling and the OXA-induced lipidomic response should be regarded as a testable modifier rather than an established resistance mechanism. Direct validation will require genetic or pharmacological perturbation of ABCA1 or SREBP2 across defined TP53 backgrounds, combined with measurements of mevalonate/cholesterol synthesis, membrane lipid composition, intracellular platinum accumulation, platinum DNA adducts, and OXA induced cell death.

FAO represents a mechanistically distinct lipid dependent route that may reinforce DNA repair independently of the p53–ABCA1–SREBP2 axis. Following DNA damage, FAO derived acetyl-CoA can promote PARP1 acetylation, PARylation, and homologous recombination, whereas FAO inhibition reduces PARP1 activity and increases DNA damage (20). Evidence that FAO supports PARP1-dependent DNA repair derives mainly from ionizing-radiation and doxorubicin models; its contribution to OXA resistance therefore requires direct testing in OXA-sensitive, post-treatment residual, and stably resistant CRC models.

Together, these observations suggest that p53 and lipid metabolism may shape the response to OXA through two mechanistically distinct routes: p53 dependent control of cholesterol/mevalonate biosynthesis and FAO dependent reinforcement of PARP1 mediated repair (**Figure 1A**). This model neither assumes that all TP53 variants produce the same metabolic state nor treats either route as a universal mechanism of OXA resistance. Future studies should use time resolved analyses to link defined TP53 variants and lipid flux to ABCA1 SREBP2 activity, PARP1 acetylation and PARylation, HR efficiency, platinum DNA adducts, and apoptotic output.

**Figure 1:**
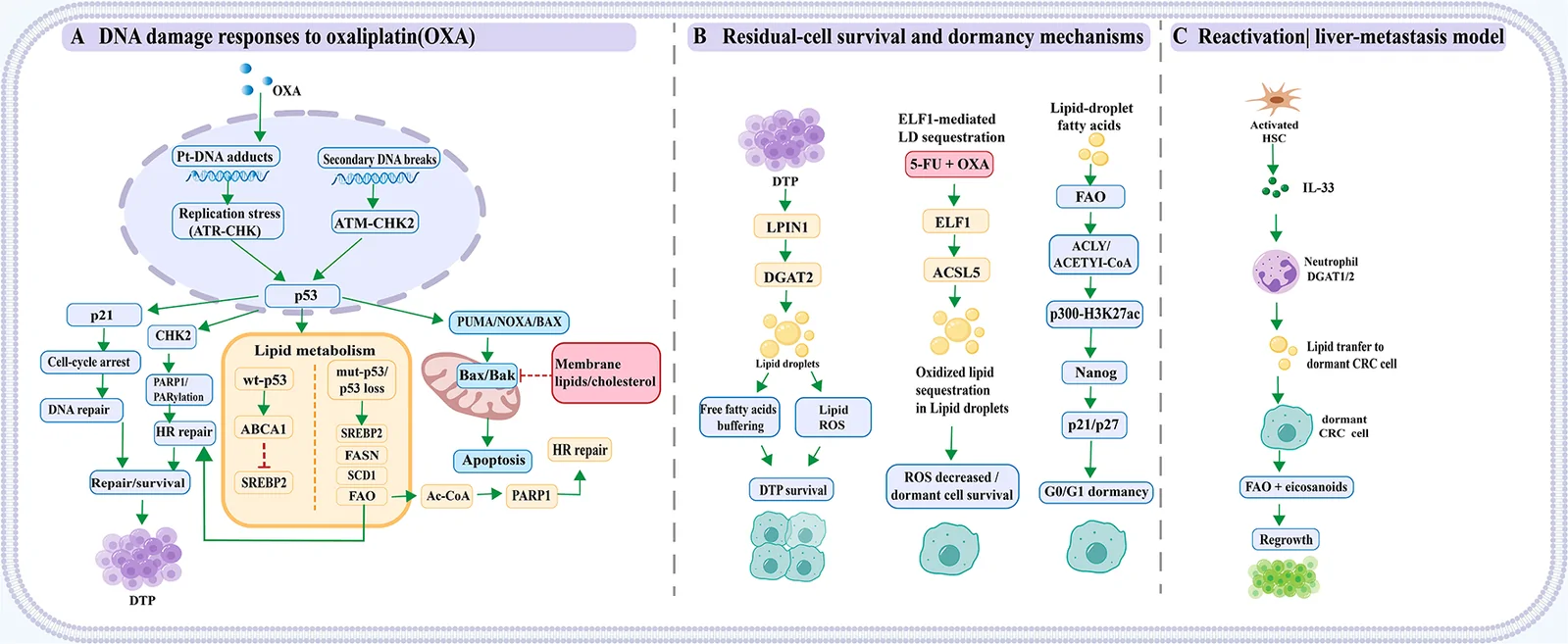
Lipid-associated regulation of DNA damage responses, residual-cell survival, and dormancy in colorectal cancer. **(A)** Oxaliplatin-induced DNA damage responses and their connections to p53-associated lipid metabolism, DNA repair, and apoptosis. **(B)** Lipid-droplet formation, ELF1–ACSL5-mediated sequestration of oxidized lipids under combined 5-FU/oxaliplatin exposure, and fatty acid oxidation-associated pathways supporting residual-cell survival or dormancy. **(C)** IL-33-associated lipid transfer from neutrophils promotes dormant-cell reactivation in a liver-metastasis model. Green solid arrows indicate activation, promotion, or process direction; red dashed blunt-ended lines indicate inhibition. Gray vertical dashed lines separate panels, while the orange dashed line separates the wild-type p53 and mutant-p53/p53-loss branches. Colored boxes and shaded areas distinguish functional groups or schematic compartments and do not indicate evidence strength.

## 3 Lipid metabolic regulation of OXA induced ferroptosis

Ferroptosis is a form of regulated cell death driven by uncontrolled iron dependent phospholipid peroxidation. Its execution depends on the sustained oxidation of membrane phospholipids containing polyunsaturated fatty acids (PUFA-PLs), which ultimately compromises membrane integrity. Although the canonical mechanism of OXA involves the formation of platinum–DNA adducts, studies in selected CRC models have shown that OXA can also increase ROS production and lipid peroxidation. Ferroptosis inhibitors partially attenuate OXA induced cytotoxicity, suggesting that ferroptosis contributes to OXA mediated cell killing but is not the sole mode of cell death across all cellular contexts. Whether CRC cells cross the threshold for ferroptosis is largely determined by the availability of PUFA-PL substrates, their capacity to eliminate lipid peroxides, and the distribution of lipids between cellular membranes and lipid droplets (**Figures 2B, C**).

**Figure 2:**
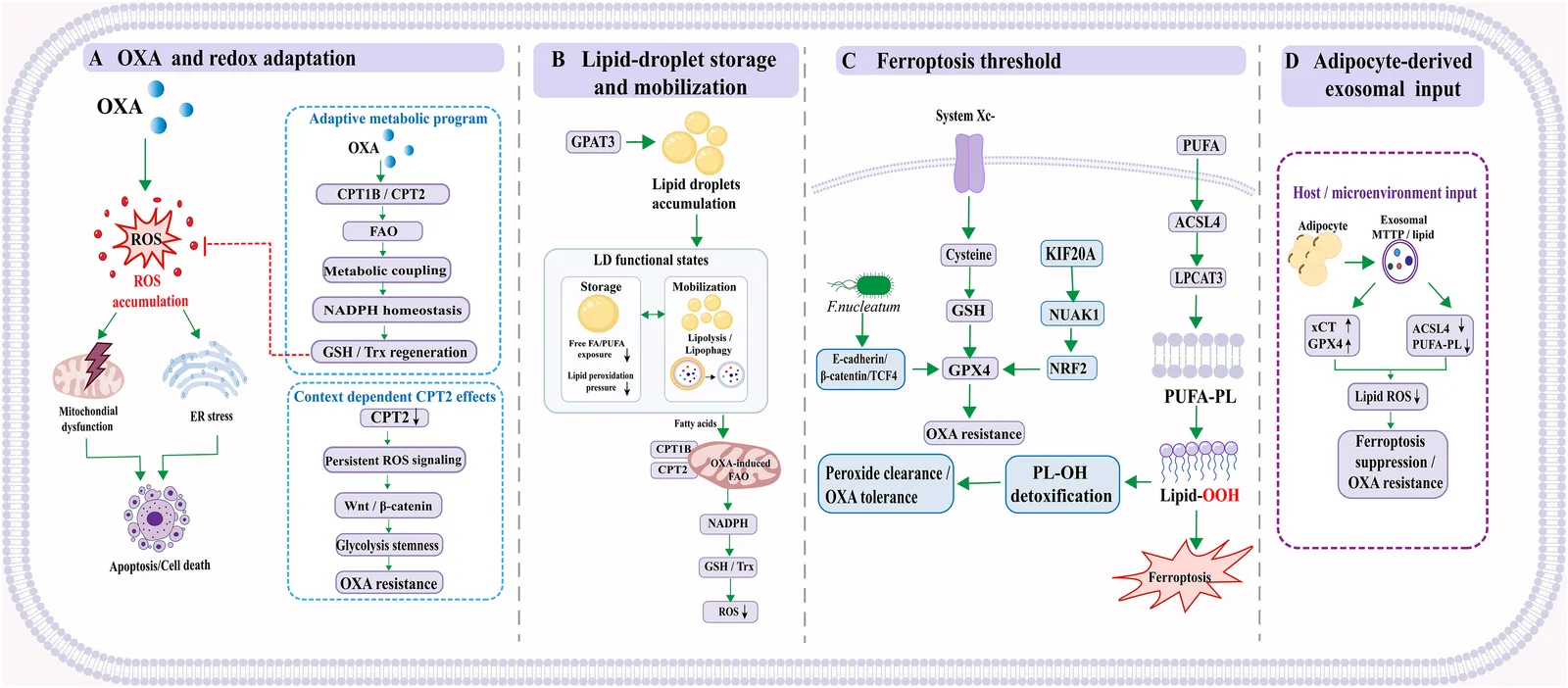
Lipid metabolic regulation of redox adaptation and ferroptosis in colorectal cancer. **(A)** Oxaliplatin-associated oxidative stress and context-dependent CPT1B/CPT2-mediated metabolic adaptation. **(B)** Lipid-droplet storage and mobilization, with proposed links to fatty acid oxidation and antioxidant defense. **(C)** The ferroptosis threshold is determined by the balance between ACSL4/LPCAT3-dependent phospholipid peroxidation and GPX4-dependent peroxide detoxification. **(D)** Adipocyte-derived exosomal MTTP promotes ferroptosis suppression through altered antioxidant defenses and lipid composition. Green solid arrows indicate activation, promotion, or process direction; red dashed blunt-ended lines indicate inhibition. Gray vertical dashed lines separate panels.Upward/downward arrows indicate increases/decreases. Colored dashed boxes group related mechanisms.

Among the molecular mediators governing lipid peroxide clearance, glutathione peroxidase 4 (GPX4) plays a pivotal role. GPX4 is a selenocysteine‑containing selenoprotein and the primary enzymatic suppressor of ferroptosis in mammalian cells. Uniquely among glutathione peroxidase family members, GPX4 directly reduces membrane‑embedded phospholipid hydroperoxides into non‑toxic lipid alcohols, thereby terminating lipid radical chain reactions and preserving plasma membrane integrity; its catalytic activity strictly depends on reduced glutathione (GSH) as a co‑substrate. Genetic or pharmacological GPX4 inactivation drives massive lipid‑peroxide accumulation and robust ferroptotic cell death across diverse cell types (21). First, the system xc^–^–GSH–GPX4 axis provides a major line of defense against OXA-induced ferroptosis. System xc^–^, composed of SLC7A11 and SLC3A2, imports cystine to sustain GSH synthesis. As a selenocysteine‑containing selenoprotein and the primary enzymatic suppressor of ferroptosis in mammalian cells, GPX4 then uses GSH to reduce phospholipid hydroperoxides, thereby interrupting lipid radical chain reactions (22). Studies of OXA resistant CRC have shown that KIF20A/NUAK1/NRF2 signaling can strengthen GPX4 dependent antioxidant defenses (23). The gut bacterium *Fusobacterium nucleatum* can likewise upregulate GPX4 through E-cadherin/β-catenin/TCF4 signaling (24). Despite their distinct origins, both pathways enhance the capacity of CRC cells to eliminate lipid peroxides, allowing them to survive the oxidative stress imposed by OXA. GPX4 therefore represents one of the best supported convergence points for ferroptosis evasion in OXA resistant CRC. In multiple human malignancies, GPX4 is frequently up‑regulated to counteract oxidative stress, enabling tumour cells to evade ferroptosis and sustain survival under chemo‑ or radiotherapy pressure. Elevated GPX4 activity has been linked to malignant phenotypes including proliferation, metastasis, and therapeutic resistance, though its biological output can be context‑dependent across different tumor subtypes (25). In CRC, increased GPX4 expression helps tumor cells withstand oxidative damage triggered by OXA treatment, representing a core mechanism for ferroptosis‑driven chemoresistance (26). Multiple upstream oncogenic and microenvironmental signals, including NRF2‑mediated antioxidant transcriptional signalling, Wnt/β‑catenin‑TCF4 axis, and KIF20A/NUAK1 pathways, converge to elevate GPX4 abundance, whereas GPX4 inhibition restores ferroptosis sensitivity in OXA‑resistant CRC pre‑clinical models.

FSP1–CoQ10 (27), DHODH–CoQ (28), and GCH1–BH4 (29) also suppress lipid peroxidation, although their roles in CRC OXA resistance have not been defined. The abundance of oxidizable substrates within membrane lipids determines whether ferroptosis can proceed. ACSL4 expression is a major determinant of cellular ferroptosis susceptibility across multiple tumor types. It is an endoplasmic‑reticulum‑localized lipid‑activating enzyme that catalyzes the ligation of long‑chain PUFAs, including arachidonic acid and adrenic acid, and cooperates with enzymes such as LPCAT3 to facilitate their incorporation into membrane phospholipids,which constitute the primary peroxidizable lipid substrates required for ferroptosis execution (30). Direct evidence from CRC models shows that CDK1 promotes ACSL4 degradation, thereby reducing PUFAs containing phospholipids and lipid peroxidation, suppressing ferroptosis, and promoting OXA resistance. Conversely, CDK1 inhibition or restoration of ACSL4 increases sensitivity to OXA (31). These findings demonstrate that a general increase in ROS does not, by itself, indicate ferroptosis. Lipid peroxidation can reach lethal levels only when oxidative stress acts on a sufficiently large pool of PUFA-PLs and the capacity of lipid peroxide–detoxifying systems is inadequate. Lipid droplets (LDs) further shape the intracellular distribution of PUFAs. When incorporated into membrane phospholipids, PUFAs expand the pool of oxidizable substrates required for ferroptosis. By contrast, their conversion into triacylglycerols and sequestration within LDs may temporarily reduce the abundance of oxidizable lipids in cellular membranes. However, lipolysis and lipophagy can remobilize stored fatty acids. LDs may therefore protect cells under conditions of lipid storage but promote lipid peroxidation when their contents are mobilized (32). GPAT3 mediated LD accumulation has been linked to chemoresistance in CRC, but current evidence does not demonstrate that GPAT3 promotes OXA resistance by suppressing ferroptosis (33). Thus, current evidence only suggests that GPAT3 mediated lipid droplet homeostasis may modulate the susceptibility of CRC cells to ferroptosis during OXA treatment; it remains insufficient to establish GPAT3 as a definitive mechanism of ferroptosis evasion.

Host metabolism and the tumor microenvironment can also modulate this process. Studies of adipocyte derived exosomal MTTP provide a relatively direct link between obesity, lipid metabolism, ferroptosis, and OXA resistance (**Figure 2D**). Canonically, MTTP exerts its physiological function within the endoplasmic reticulum to transfer triglycerides and participate in lipoprotein assembly. However, under an obese tumor microenvironment, functional MTTP protein is packaged into exosomes secreted by adipocytes and horizontally transferred to recipient CRC cells, where it rewrites intracellular lipid homeostasis of tumour cells. Notably, MTTP is not an intrinsic drug‑resistance factor overexpressed by tumour cells themselves. Host metabolism and the tumour microenvironment can also modulate this process. Studies on adipocyte‑derived exosomal MTTP provide relatively direct mechanistic evidence linking host adiposity, intercellular lipid‑related signalling, ferroptosis suppression and OXA resistance (34). After exosome‑delivered MTTP is internalized by CRC, it forms a complex with PRAP1 to repress ZEB1 transcription. This MTTP‑PRAP1‑ZEB1 signalling cascade triggers two major lipid‑associated alterations: it strengthens the xCT/GPX4‑dependent anti‑ferroptotic antioxidant defence system and suppresses ACSL4 expression, which collectively elevates xCT and GPX4 levels, decreases the proportion of PUFAs and lipid ROS, and consequently promotes OXA resistance in patient‑derived organoids and obese mouse models (11). Down‑regulated ACSL4 further diminishes the cellular pool of polyunsaturated fatty acid‑containing phospholipids (PUFA‑PLs), alleviates lipid‑ROS burden, and blunts oxaliplatin‑triggered ferroptosis. This lipid‑reprogramming phenotype confers oxaliplatin resistance, and this effect has been validated in patient derived colorectal cancer organoids and obese tumour bearing mouse models (9). Notably, chemotherapeutic stress can further promote MTTP secretion from adipocytes, creating a positive feedback vicious cycle that amplifies drug resistance (35). The above mentioned evidence is mainly derived from pre‑clinical organoid and animal experiments. At present, prospective human clinical trial data targeting exosomal MTTP to reverse oxaliplatin resistance remain lacking.These findings indicate that escape from OXA induced ferroptosis is not determined solely by tumor cell intrinsic metabolism. Adipose tissue can also alter PUFAs substrate availability and antioxidant defenses through exosomal signaling. Similarly, the finding that *Fusobacterium nucleatum* upregulates GPX4 suggests that signals from the gut microbiota can converge on lipid antioxidant programs within tumor cells (24).

p53 may serve as an interface between the DNA damage response and ferroptosis, but these processes cannot currently be placed within a single, established unidirectional pathway (36–38).The prognostic relevance of ferroptosis related molecules should not be equated with their ability to predict the therapeutic efficacy of OXA. Their significance must instead be evaluated through an integrated assessment of PUFA-PL abundance, lipid droplet burden, iron levels, lipid ROS, and antioxidant defenses.

Future studies should collect serial paired samples from the same patients or experimental models before OXA treatment, during the early treatment response, at the post-treatment residual cell stage, and after stable acquired resistance has emerged. These samples should be analyzed for ACSL4/LPCAT3 expression, the PUFA-PL lipidome, lipid droplet burden, the SLC7A11–GSH–GPX4 axis, NRF2, the FSP1–CoQ10 system, and lipid ROS. These measurements should also be integrated with changes in patient adiposity, circulating lipid levels, glucose metabolism, the gut microbiota, and ctDNA/MRD. Only by distinguishing pre-existing metabolic states from OXA induced reversible adaptations and stable drivers of resistance will it be possible to identify the appropriate patient populations and treatment windows for combining OXA with ferroptosis inducers, PUFA based interventions, or lipid metabolism targeting agents.

## 4 Lipid metabolism limits OXA induced ROS accumulation

The accumulation of ROS is an important contributor to the cytotoxic effects of OXA. In addition to forming platinum–DNA adducts, OXA can disrupt mitochondrial function, induce endoplasmic reticulum stress, and increase intracellular ROS levels. When ROS accumulation exceeds the detoxification capacity of cellular antioxidant systems, it can damage DNA, proteins, and membrane lipids, thereby triggering apoptosis or other forms of regulated cell death. However, CRC cells can preserve redox homeostasis through lipid metabolic reprogramming, preventing OXA induced ROS from reaching a lethal threshold. Among these adaptations, FAO and its dynamic interplay with lipid droplets are supported by relatively strong evidence.FAO involves the transport of long chain fatty acids into mitochondria, where they undergo β-oxidation to generate acetyl-CoA, NADH, and FADH_2_ and support ATP production. Importantly, β-oxidation does not directly generate NADPH. However, by sustaining mitochondrial metabolism and associated metabolic coupling pathways, it can help maintain cellular NADPH homeostasis (10). NADPH, in turn, supports the regeneration of the glutathione and thioredoxin systems, thereby enhancing the cellular capacity to eliminate ROS. Increased FAO may therefore both provide energy to damaged cells and maintain the supply of reducing equivalents, helping CRC cells tolerate OXA induced oxidative stress (**Figure 2A**). A mechanistic study performed in *in vitro* models showed that OXA treatment increased the expression of fatty acid catabolism related enzymes, including CPT1B and CPT2, in gastric cancer and CRC cells. Clinical correlative analyses of gastrointestinal cancer patient samples further revealed an association between high CPT1B/CPT2 expression and poorer outcomes following OXA‑based chemotherapy; this represents a prognostic correlation rather than causal proof.Genetic inhibition of CPT2 or pharmacological blockade of fatty acid catabolism with perhexiline disrupted NADPH and redox homeostasis, increased OXA induced ROS accumulation and apoptosis, and enhanced the antitumor activity of OXA in cell derived xenograft and patient derived xenograft models (13). These findings suggest that increased FAO following OXA exposure may help CRC cells withstand OXA induced oxidative damage by sustaining cellular reducing power and limiting excessive ROS accumulation, thereby contributing to a treatment induced state of adaptive tolerance. Notably, these observations are derived from pre‑clinical *in vitro* and *in vivo* systems and cannot be directly extrapolated to human patients. However, the sensitizing effect of combining FAO inhibition with OXA is currently supported largely by cell based and animal studies and has not been clinically validated in patients with CRC. These preclinical findings therefore should not be interpreted as evidence that this strategy improves patient survival.

The role of CPT2 in CRC has also yielded apparently conflicting findings. Another study reported reduced CPT2 expression in a subset of CRC tissues, with low CPT2 levels associated with advanced disease, poor prognosis, and OXA resistance. Restoration of CPT2 expression reduced ROS levels, suppressed Wnt/β-catenin signaling, glycolysis, and tumor cell stemness, and increased sensitivity to OXA (39). These observations were obtained from *in vitro* CRC cell line experiments and clinical patient‑tissue correlative analysis. These findings do not imply that lowering ROS necessarily sensitizes CRC cells to OXA. Rather, they indicate that sustained ROS elevation can also act as a signaling stimulus that activates Wnt/β-catenin and stemness programs, thereby establishing a distinct state of drug resistance.The two studies were conducted in different experimental contexts. The former examined the short term induction of CPT1B/CPT2 and adaptive FAO following OXA exposure, whereas the latter focused on pre-existing or chronically acquired CPT2 downregulation accompanied by the reprogramming of glycolysis and tumor cell stemness. CPT2 therefore cannot be classified simply as either a promoter or a suppressor of OXA resistance. A more plausible interpretation is that the response to OXA depends on basal CPT2 expression, its dynamic changes after treatment, the cellular metabolic context, and the duration of ROS exposure. However, this time dependent model represents an integrative inference synthesized from multiple independent datasets and requires longitudinal validation within the same experimental system.

LDs may provide an important structural link between lipid storage and adaptive FAO. Following OXA induced oxidative stress, cells can esterify free fatty acids and readily oxidizable polyunsaturated fatty acids into triacylglycerols and sequester them within LDs, temporarily reducing free fatty acid toxicity and membrane lipid peroxidation. Under sustained drug pressure, LDs can subsequently release fatty acids through lipolysis or lipophagy, thereby supplying substrates for mitochondrial FAO (32). LDs accumulation in drug tolerant cells does not necessarily indicate that lipids are held in a static storage state; it may instead reflect simultaneous increases in lipid droplet formation and mobilization. This enhanced turnover could establish a dynamic metabolic cycle in which fatty acids are initially sequestered in lipid droplets and subsequently mobilized and channeled into FAO to support cellular energy production and maintain reducing power (**Figure 2B**). However, studies that longitudinally measure LD biogenesis, lipolysis, FAO flux, NADPH, and ROS within the same CRC model are currently lacking. This cycle should therefore not yet be described as a fully established mechanism of OXA resistance. The GPAT3 study provides relatively direct pre‑clinical evidence from *in vitro* cell line and *in vivo* xenograft models that lipid droplets contribute to OXA tolerance. GPAT3 promotes triacylglycerol synthesis and lipid droplet formation, and its overexpression increases the tolerance of CRC cells to OXA, whereas inhibition of lipid droplet formation enhances OXA induced cell death. The study also showed that GPAT3 mediated lipid droplet accumulation reduced cell surface calreticulin exposure and weakened CD8^+^ T cell responses (33), suggesting that lipid droplets influence both tumor cell survival and OXA induced immunogenic cell death (**Figure 2B**). However, the study did not directly demonstrate that GPAT3 facilitates ROS clearance by enhancing FAO or increasing NADPH availability. Current evidence therefore supports only that GPAT3 mediated lipid droplet accumulation promotes tolerance to OXA. Whether this effect is jointly driven by lipid droplet mobilization, increased FAO, and enhanced ROS buffering remains unresolved.

More recent evidence identifies an ELF1-ACSL5-lipid-droplet axis that links lipid partitioning to redox adaptation in chemotherapy-induced dormant CRC cells. In PDX-derived CRC cells and LoVo cells exposed to 5-FU plus OXA, dormant cells accumulated lipid droplets and showed increased ACSL5 expression. ELF1 transcriptionally activated ACSL5, whereas ACSL5 promoted the sequestration of oxidized lipids within lipid droplets, thereby lowering intracellular ROS. Genetic ACSL5 depletion or pharmacological inhibition of DGAT1/2 disrupted this sequestration, increased ROS, and reduced the dormancy markers NR2F1 and p27; conversely, ACSL5 overexpression suppressed ROS, and this effect was lost when lipid-droplet formation was inhibited. These results demonstrate that lipid-droplet compartmentalization can buffer oxidative stress and sustain residual CRC-cell survival (40). However, because dormancy was induced with combined 5-FU/OXA rather than OXA alone, the ELF1-ACSL5 axis should be interpreted as a fluoropyrimidine-OXA treatment-context mechanism rather than an OXA-specific resistance pathway.

Future studies should longitudinally assess lipid droplet abundance and turnover, CPT1B/CPT2 expression, FAO flux, NADPH/NADP^+^ and GSH/GSSG ratios, and ROS levels before OXA treatment, during the early response, and after stable resistance has developed. FAO activity should not be inferred solely from the expression of a single enzyme. Only by determining whether lipid droplet accumulation represents static storage or a high turnover state, and whether altered FAO reflects a treatment induced adaptation or a pre-existing metabolic feature, will it be possible to establish whether inhibiting lipid droplet biogenesis or blocking FAO is an appropriate strategy for sensitizing CRC to OXA.

## 5 Lipid metabolism and tumor dormancy

Tumor dormancy refers to a reversible state of low proliferative activity that tumor cells enter in response to therapeutic or microenvironmental stress, with the capacity to resume proliferation when conditions become favorable. In patients with CRC, small numbers of circulating tumor cells or microscopic disseminated lesions may persist after curative intent resection even when no metastases are detectable by imaging. Adjuvant FOLFOX or CAPOX is intended to eradicate these residual cells; however, some cells may evade chemotherapy by entering a quiescent or DTP state, thereby providing a potential cellular source of minimal residual disease and late recurrence (4, 41). DTP states, cellular dormancy, and clinical minimal residual disease are related but not equivalent concepts. Findings obtained from quiescent cells *in vitro* or *ex vivo* models therefore cannot be directly extrapolated to explain recurrence in patients.

In 2025, Morshneva et al. established an *in vitro* recurrence model using platinum resistant HCT116 cells. OXA treatment increased the proportion of quiescent cells arrested in G0/G1. The surviving cells showed increased expression of stemness markers, lower ROS levels, enhanced autophagy, and greater tolerance to chemotherapy, and resumed proliferation during subsequent culture. The model recapitulated the transition of residual cells from post treatment quiescence to regrowth (42). Because this model used cells with pre-existing platinum resistance, it shows that OXA can enrich or maintain dormancy like cells in a resistant background rather than proving *de novo* dormancy induction in treatment naive CRC.

Residual tumor cells are not metabolically inert. Although their proliferation and some biosynthetic activities are reduced, the maintenance of membrane integrity, redox homeostasis, and long term survival still requires selective metabolic support. Lipid droplets may serve as lipid buffers in this setting: the conversion of free fatty acids into triacylglycerols and their storage within lipid droplets can limit free fatty acid toxicity and lipid peroxidation. Conversely, when energy or membrane lipid precursors become limiting, lipid droplets can release fatty acids for cellular use (32). Lipid droplets may therefore function both as protective storage depots and as metabolic reserves that support the long term survival and eventual reentry of dormant cells into proliferation.The most direct evidence linking lipid droplets to residual CRC cells currently comes from studies of LPIN1 performed across *in vitro* DTP cell models, *ex vivo* patient derived organoids, and *in vivo* CDX/PDX xenograft models. CRC DTP cells, including persister cells generated following combined 5-FU and OXA treatment, exhibit increased LPIN1 and DGAT2 expression together with marked lipid droplet accumulation. LPIN1 catalyzes the conversion of phosphatidic acid into diacylglycerol, after which DGAT2 promotes triacylglycerol synthesis and lipid droplet formation, allowing excess free fatty acids to be sequestered within neutral lipid stores. Inhibition of LPIN1 or disruption of this metabolic process with statins reduces lipid droplet accumulation, increases lipotoxicity and lipid ROS, and induces ferroptosis. These effects have been demonstrated in DTP cells, patient derived organoids, cell derived xenografts, and patient derived xenograft models (43). These findings indicate that lipid droplets are not merely an accompanying phenotype of DTP cells but may represent functionally important structures that maintain lipid homeostasis and support long term survival. These data establish a role for LPIN1-dependent LD formation in DTP cell survival, but not in OXA specific clinical dormancy: the models included 5-FU or irinotecan, LPIN1 inhibition primarily induced lipotoxicity and ferroptosis, and statins are not LPIN1 specific. Accordingly, LD disruption remains an experimental DTP targeting strategy rather than a basis for routine statin use in CRC.

In addition to lipid droplet storage, FAO may contribute to the establishment of a dormant phenotype. A study *in vitro* CRC cell line models showed that serum deprivation induced reversible G0/G1 cell cycle arrest. During this process, FAO increased ACLY expression and acetyl-CoA production, thereby promoting p300 mediated H3K27 acetylation at the Nanog promoter and enhancing Nanog transcription. Nanog subsequently upregulated molecules including p21 and p27, helping cells maintain a low proliferative state. Inhibition of FAO, ACLY, or p300 weakened this dormant phenotype and increased cell death (44). The role of FAO may therefore extend beyond energy production. FAO may promote ACLY dependent acetyl-CoA production, thereby enhancing p300 mediated H3K27 acetylation and Nanog transcription. The resulting upregulation of p21 and p27 may help maintain a low proliferative dormant state through epigenetic regulation. Because this study used serum deprivation rather than OXA exposure, it provides mechanistic evidence that lipid metabolism can regulate dormancy in CRC but does not demonstrate that OXA induces dormancy through this axis. Moreover, the functional continuum between lipid droplets and FAO has not been fully validated within the same model of OXA surviving residual cells. It remains unclear whether fatty acids stored in lipid droplets are continuously mobilized into FAO and whether FAO blockade causes dormant cell death or instead drives premature exit from dormancy and renewed proliferation.

Reactivation of dormant tumor cells may also depend on exogenous lipids supplied by the metastatic microenvironment. A 2025 study of CRC liver metastasis combined *in vitro* co‑culture assays and *in vivo* mouse models. Activated showed that activated hepatic stellate cells secrete IL33, which promotes lipid droplet synthesis in mature low density neutrophils through mTOR/PPARγ signaling and DGAT1/2. Following uptake of these neutrophil derived lipids, dormant tumor cells increase β-oxidation and the synthesis of protumorigenic eicosanoids, thereby resuming proliferation. In mouse models, IL33 blockade reduced lipid accumulation in neutrophils and decreased hepatic colonization by CRC cells (45). Overall, the available evidence supports a model in which lipid metabolism may shape the fate of residual CRC cells through three sequential stages. First, LPIN1/DGAT2 mediated lipid droplet formation buffers lipotoxicity and sustains the survival of DTP cells (**Figure 1B**). Second, FAO/ACLY/Nanog signaling may stabilize a low proliferative state (**Figure 1B**). Finally, exogenous lipids supplied by cells in the microenvironment may trigger dormant cell reactivation through FAO and eicosanoid synthesis (**Figure 1C**). By contrast, the reported associations of serum triglycerides, apolipoprotein B, high density lipoprotein cholesterol, and FABP4 with recurrence or survival remain largely clinical correlative observations and do not establish that these factors directly maintain dormant cells after OXA treatment.Future studies should longitudinally assess cell cycle status, LPIN1/DGAT2 expression, lipid droplet turnover, FAO flux, ACLY/Nanog signaling, and the sources of cellular lipids at four stages: before OXA treatment, during the early residual phase, during quiescence after treatment withdrawal, and upon renewed proliferation. These analyses should be performed using patient derived organoids and integrated with longitudinal monitoring of ctDNA and MRD. Only by determining whether lipid droplets and FAO regulate entry into dormancy, maintenance of the dormant state, or subsequent reactivation can we establish whether they are merely prognostic biomarkers or actionable therapeutic targets for eliminating residual cells and reducing the risk of recurrence.

## 6 Lipid metabolism and the tumor microenvironment

In addition to forming platinum–DNA adducts and directly killing tumor cells, OXA can induce immunogenic cell death (ICD). Following OXA treatment, CRC cells may expose calreticulin (CRT) on their surface, release ATP, and discharge high mobility group box 1 (HMGB1) into the extracellular environment. As an”eat myself” signal, surface exposed CRT promotes the recognition and uptake of dying tumor cells by dendritic cells. Extracellular ATP recruits and activates antigen presenting cells, whereas HMGB1 contributes to antigen processing and inflammatory signaling. Together, these damage associated molecular patterns help initiate tumor specific CD8^+^ T cell responses, extending the effects of OXA beyond direct cytotoxicity to the activation of antitumor immunity (46).The ability to induce ICD is not shared by all platinum based agents. A landmark study in colon cancer models showed that both OXA and cisplatin caused DNA damage and induced HMGB1 release, whereas OXA was substantially more effective at promoting pre-apoptotic CRT exposure. This distinction may arise because OXA links DNA damage to endoplasmic reticulum stress, activation of the PERK–eIF2α phosphorylation pathway, and the translocation of CRT from the endoplasmic reticulum to the plasma membrane. Cisplatin alone has a relatively limited capacity to induce CRT exposure, although its immunogenicity can be partially restored when combined with an inducer of endoplasmic reticulum stress (46).Thus, OXA induced ICD is not an inevitable consequence of platinum DNA adduct formation but depends on the integrity of the endoplasmic reticulum stress response and danger signal release machinery.

CRT, ATP, and HMGB1 indicate ICD-related signaling, but effective tumor control additionally requires competent dendritic cells, tumor antigenicity, CD8^+^ T-cell infiltration, and limited myeloid immunosuppression. For example, M2 like tumor-associated macrophages can induce METTL3 expression in CRC cells, promoting the m6A modification and degradation of TRAF5 mRNA, thereby suppressing necroptosis and increasing OXA resistance (8). Although this study did not investigate a lipid metabolic mechanism, it demonstrates that the immune microenvironment can actively reshape the mode of cell death induced by OXA, rather than merely responding passively to tumor cell death. Among lipid metabolic factors, lipid droplet accumulation currently provides the most direct mechanistic link between OXA resistance and impaired ICD. Lipid droplets are dynamic organelles that store triglycerides and cholesteryl esters. In addition to supplying lipids and buffering oxidative stress, they can influence endoplasmic reticulum homeostasis and cell death signaling. Lysophosphatidylcholine acyltransferase 2 (LPCAT2) is a lipid droplet localized metabolic enzyme involved in phosphatidylcholine synthesis. Across different CRC cell lines, basal lipid droplet content was positively correlated with LPCAT2 expression and the half maximal inhibitory concentrations of both 5-fluorouracil and OXA. Mechanistically, LPCAT2 mediated lipid droplet accumulation attenuated chemotherapy induced endoplasmic reticulum stress and caspase cascade activation, thereby reducing tumor cell death (47). Importantly, this study linked lipid droplets to the immune effects induced by OXA. Increased lipid droplet content was associated with reduced cell surface CRT exposure and attenuated ICD, accompanied by decreased CD8^+^ T cell infiltration in mouse tumor models. Analysis of metastatic lesions from patients with CRC likewise revealed an inverse association between lipid droplet accumulation and CD8^+^ T cell infiltration (47). These findings suggest that tumors with a high lipid droplet burden may not only better withstand the direct cytotoxic effects of OXA but may also be less able to convert tumor cell death into effective immune recognition. Patient-tissue data support an inverse association between LD burden and CD8^+^ T-cell infiltration, not a causal link between LPCAT2/LD accumulation and clinical FOLFOX or CAPOX resistance.

The GPAT3 study provides further support for this mechanism. GPAT3 functions in the glycerol-3-phosphate pathway, promoting the production of lysophosphatidic acid and, subsequently, triglycerides and lipid droplets. OXA treatment increased GPAT3 expression in CRC cells, whereas GPAT3 overexpression promoted lipid droplet accumulation, reduced OXA induced apoptosis, and increased cellular resistance. Inhibition of lipid droplet formation partially restored the cytotoxic effects of OXA. At the immune level, GPAT3 mediated lipid droplet accumulation reduced cell surface CRT exposure, rendering OXA treated CRC cells poorly immunogenic. In mouse models, this alteration was accompanied by a weaker cytotoxic IFN-γ response and an increase in PD-1^+^TIM-3^+^CD8^+^ T cells, consistent with impaired CD8^+^ T cell function and enhanced exhaustion (33). Based on the findings, it is plausible to hypothesize a relatively coherent mechanistic model: OXA induces endoplasmic reticulum stress and the translocation of CRT to the plasma membrane, thereby promoting ICD and CD8^+^ T cell responses. By contrast, upregulation of LPCAT2 or GPAT3 drives lipid droplet accumulation, which buffers endoplasmic reticulum stress and attenuates caspase activation and cell surface CRT exposure. The resulting reduction in the immunogenicity of dying tumor cells limits CD8^+^ T cell recruitment and effector function, thereby allowing residual tumor cells to survive more readily (**Figure 3A**). However, the direct molecular mechanism by which lipid droplets suppress CRT translocation remains unclear. Lipid droplets may alter the lipid composition of the endoplasmic reticulum membrane, the intensity of endoplasmic reticulum stress, oxidative damage, or the execution of cell death signaling; these possibilities have not yet been fully distinguished within the same OXA model. Moreover, studies of GPAT3 and LPCAT2 have relied mainly on cell based experiments, xenograft models, and correlative analyses of patient tissues. No prospective clinical trial has established their ability to predict response to OXA, and there is currently no evidence to support the routine addition of lipid droplet inhibitors to OXA based therapy in unselected patients with CRC. Reduced cholesterol biosynthesis, lipid raft reorganization, and activation of TGF-β/SMAD–SERPINE1 signaling in OXA resistant CRC cells primarily reflect tumor cell acquired resistance and prometastatic signaling (48). By contrast, dysregulated cholesterol or fatty acid metabolism within CD8^+^ T cells may impair their effector function and promote exhaustion. Because these alterations occur in different cell types and subcellular compartments, there is currently insufficient evidence to conclude that they constitute a unified mechanism of OXA resistance.

**Figure 3:**
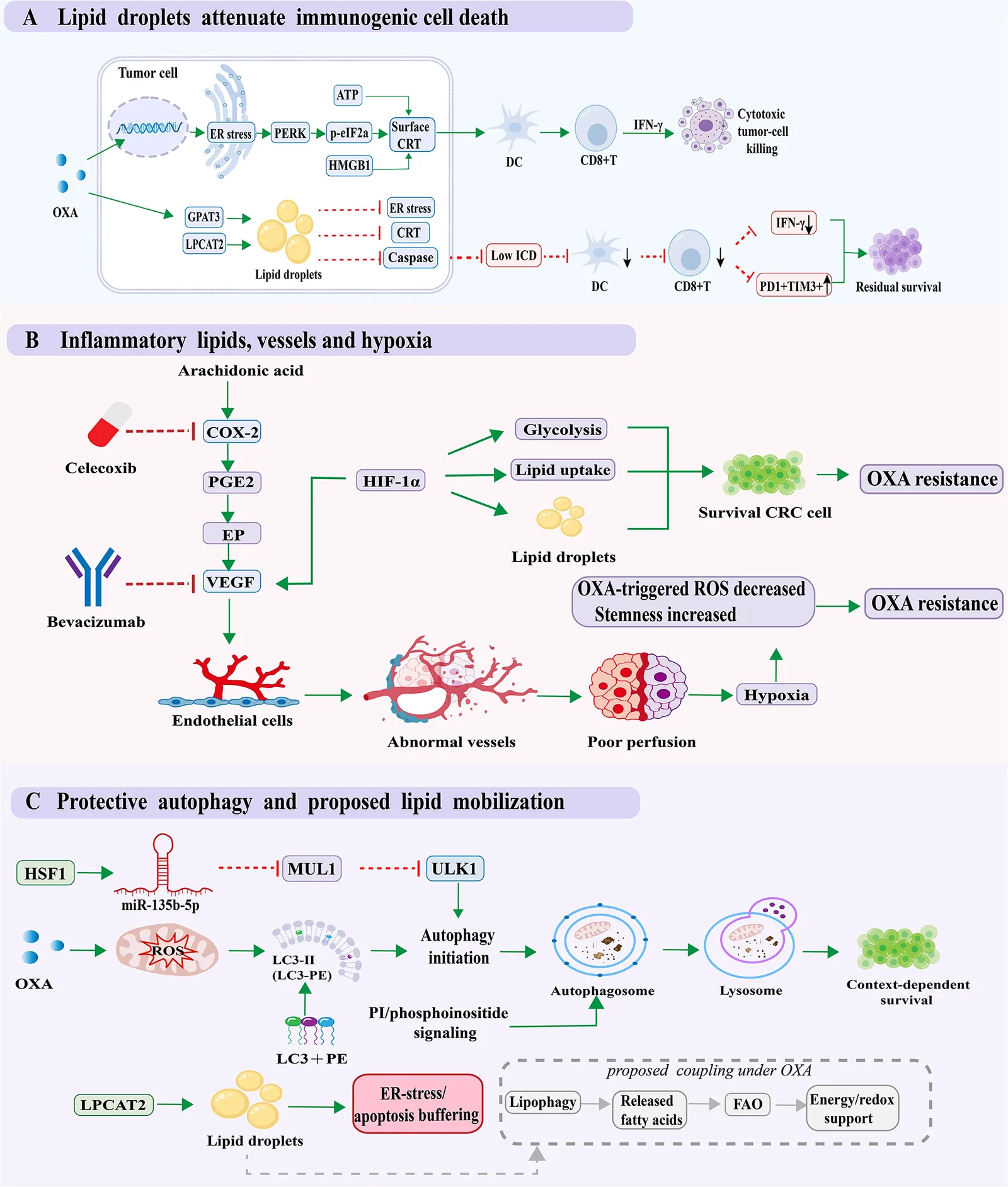
Lipid-associated regulation of antitumor immunity, vascular adaptation, and autophagy in oxaliplatin resistance. **(A)** GPAT3/LPCAT2-associated lipid droplets attenuate immunogenic cell death and antitumor immune responses. **(B)** Inflammatory lipid signaling, abnormal vasculature, and hypoxia support metabolic adaptation and tumor-cell survival. Celecoxib and bevacizumab can inhibit angiogenesis, thereby assisting OXA in reversing drug resistance. **(C)** Protective autophagy and the proposed coupling of lipophagy mediated fatty acid release to fatty acid oxidation. Green solid arrows indicate activation, promotion, or process direction; red dashed blunt-ended lines indicate inhibition. Upward/downward arrows indicate increases/decreases. Gray arrows and dashed outlines denote proposed mechanistic connections requiring direct validation. Background and label colors aid visual grouping and do not indicate evidence strength.

Beyond lipid‑droplet‑driven impairment of immunogenic cell death, chronic inflammatory signaling within the tumor microenvironment shapes chemotherapeutic responses via bioactive lipid mediator networks, an emerging dimension that remains incompletely explored in OXA‑resistance research. Chronic tumor associated inflammation is sustained not only by canonical cytokines and chemokines but also by a balance between pro‑inflammatory eicosanoid signals and specialized pro‑resolving lipid mediators (SPMs, lipid resolvers of inflammation) that actively terminate inflammatory cascades (49). In drug‑resistant tumor niches, this homeostatic balance can be disrupted: excess pro‑inflammatory lipids (including prostaglandins such as PGE2 and leukotrienes) are produced by tumor‑associated macrophages, cancer‑associated fibroblasts and malignant epithelial cells, whereas the abundance of pro‑resolving lipid mediators (resolvins, lipoxins, protectins, maresins) is frequently diminished (50).

A loss of inflammation‑resolving capacity has recently been linked to chemoresistance across multiple tumor entities, including CRC and malignant pleural mesothelioma. In mesothelioma, chemotherapy exposure reduces SPM levels in pleural effusions while elevating prostaglandin concentrations; this skewed lipid‑mediator profile fosters a protumorigenic, immunosuppressive microenvironment associated with inferior therapeutic outcomes (51). Translating these concepts to CRC, pro‑inflammatory PGE2 signalling via the EP4 receptor blunts OXA‑triggered ROS accumulation, enriches cancer stem‑cell populations and promotes chemoresistance, whereas EP4‑receptor inhibition restores OXA sensitivity in preclinical models (52). SPMs such as resolvins can enhance clearance of chemotherapy‑derived tumor‑cell debris by phagocytes and dampen debris‑driven pro‑inflammatory cytokine release; nevertheless, direct experimental evidence demonstrating whether insufficient SPM production contributes specifically to OXA failure in CRC remains sparse, and human clinical data are still largely absent. As a pro‑inflammatory lipid, PGE2 acts within the immune compartment to suppress ICD, dendritic‑cell function and CD8^+^ T‑cell effector activity, creating mechanistic crosstalk between inflammatory lipid signalling, immunogenic cell death and OXA response. Whether SPM replacement can restore antitumor immunity and augment OXA efficacy is therefore a defined question for future mechanistic and translational studies.

Accordingly, future studies should analyze paired samples collected before and after FOLFOX or CAPOX treatment, simultaneously measuring GPAT3 and LPCAT2 expression, lipid droplet density, endoplasmic reticulum stress, CRT exposure, ATP and HMGB1 release, dendritic cell activation, CD8^+^ T cell infiltration, and markers of T cell exhaustion. These variables should then be correlated with pathological response, longitudinal changes in ctDNA and MRD, and recurrence outcomes. Only if lipid droplet alterations are shown to precede the attenuation of ICD, and inhibition of lipid droplet formation restores OXA induced antitumor immunity, can lipid droplets be regarded as functional targets for overcoming resistance rather than merely an accompanying metabolic phenotype of chemotherapy tolerant tumors.

## 7 Lipid metabolism and vascular remodeling in OXA resistance

Beyond altering energy metabolism and membrane lipid supply within CRC cells, dysregulated lipid metabolism may influence the therapeutic effects of OXA by modulating angiogenesis, tumor perfusion, and the hypoxic microenvironment. Newly formed vessels in CRC are typically disorganized, hyperpermeable, poorly covered by pericytes, and unevenly perfused. These vascular abnormalities may limit the uniform distribution of OXA into deeper tumor regions while generating hypoxic niches that promote metabolic reprogramming, maintenance of tumor cell stemness, and increased invasiveness. Consequently, a reduction in vessel density does not necessarily translate into improved drug delivery; the efficacy of OXA also depends on vascular maturity, functional perfusion, and the severity of hypoxia (53).Among lipid metabolism related mechanisms, the arachidonic acid–COX-2/PGE2–VEGF axis provides a relatively direct link between CRC angiogenesis and the response to OXA. COX-2 is a key inducible enzyme that converts arachidonic acid into prostaglandin intermediates, and its activation increases the production of lipid mediators such as PGE2. Through EP receptors and their downstream signaling pathways, PGE2 can promote VEGF expression, endothelial cell activation, and abnormal vessel formation, while also contributing to inflammation, tumor cell survival, and immune suppression. Thus, COX-2/PGE2 is not merely an inflammatory signal but may represent an important interface linking lipid metabolism to remodeling of the tumor vascular microenvironment.

In an HT-29 xenograft model in nude mice, OXA monotherapy inhibited tumor growth but increased COX-2 and VEGF expression in tumor tissues after treatment, suggesting that residual tumors may develop a compensatory proangiogenic response. The selective COX-2 inhibitor celecoxib reduced VEGF expression and microvessel density. When combined with OXA, celecoxib further enhanced tumor growth inhibition and apoptosis, producing greater effects than either agent alone (54). These findings support a possible mechanism whereby OXA kills tumor cells while simultaneously inducing COX-2/VEGF signaling through inflammatory or stress responses, thereby providing vascular and microenvironmental support for residual cells. Inhibition of COX-2 may attenuate this compensatory response and enhance the antitumor effects of OXA. However, this study did not conclusively establish PGE2 as the sole mediator between COX-2 and VEGF, nor did it directly measure cholesterol levels, fatty acid synthesis, or OXA concentrations in tumor tissues. Accordingly, COX-2 inhibition was associated with lower VEGF expression and microvessel density and with enhanced tumor suppression by OXA. Nevertheless, the hypothesis that the COX-2/PGE2/VEGF axis impairs OXA delivery requires further validation through PGE2 quantification, EP-receptor perturbation, measurement of tissue platinum content, and assessment of tumor perfusion. In addition, celecoxib can affect cell cycle progression, apoptosis, and other COX-2-independent targets; therefore, the benefit of the combination cannot be attributed entirely to its antiangiogenic activity.

Vascular abnormalities may sustain a tumor microenvironment that favors tolerance to OXA through a hypoxia driven feedback loop. Inadequate perfusion stabilizes HIF-1α, which in turn induces the expression of proangiogenic factors such as VEGF. However, the resulting neovessels are typically structurally disorganized and poorly perfused and therefore fail to alleviate hypoxia, reinforcing both vascular dysfunction and the hypoxic state.At the same time, HIF-1α promotes glycolysis, lipid uptake, and lipid droplet formation, enabling tumor cells to survive under hypoxic conditions and fluctuating nutrient availability. Experimentally, hypoxia reduced the sensitivity of CRC cells, including HCT116 cells, to OXA. A metabolic intervention that promoted HIF-1α degradation increased OXA-induced cell death and reduced VEGFR2 and CD31 levels in tumors from treated animals (7). These findings position HIF-1α between metabolic adaptation, angiogenesis, and OXA resistance, while the requirement for LDs or FAO remains untested. Studies of antiangiogenic therapy further indicate that reducing tumor vessel density does not necessarily translate into improved therapeutic efficacy. In an HCT116 xenograft model, bevacizumab reduced microvascular indices but increased vasculogenic mimicry, in which tumor cells form lumen like blood supply channels without the involvement of conventional endothelial cells. Combining OXA with bevacizumab produced greater tumor growth inhibition and partially restricted this alternative vascular adaptation (55). These findings suggest that tumors may maintain their nutrient supply through vasculogenic mimicry and related compensatory mechanisms after VEGF blockade. The efficacy of combination chemotherapy therefore depends not only on reducing neovascularization but also on limiting the invasiveness and compensatory blood supply capacity of residual tumor cells.However, this study did not directly examine lipid metabolism and therefore provides only contextual evidence that remodeling of the tumor blood supply can influence OXA efficacy; it does not demonstrate that lipid metabolism drives vasculogenic mimicry. Similarly, studies linking FASN expression to VEGF or endothelial cell activation support a role for fatty acid synthesis in CRC angiogenesis, but they have not established that FASN promotes OXA resistance through vascular remodeling. Studies examining FASN genetic polymorphisms and bevacizumab efficacy were conducted mainly in patients receiving FOLFIRI plus bevacizumab. Although their findings suggest that lipid metabolism may influence the response to anti VEGF therapy, they cannot be directly extrapolated to FOLFOX or CAPOX.

Accordingly, the available evidence supports a mechanistic framework centered on COX-2 and hypoxia (**Figure 3B**). OXA induced stress or tumor associated inflammation promotes the production of COX-2 related lipid mediators and activates VEGF signaling, thereby helping to maintain abnormal tumor vasculature. The resulting inadequate perfusion stabilizes HIF-1α, which enhances metabolic adaptation and tolerance to OXA. Although anti VEGF therapy can suppress conventional angiogenesis, it may also exacerbate hypoxia or induce alternative modes of tumor blood supply. Therefore, lipid metabolism may serve as a critical node linking inflammatory mediator production, proangiogenic signaling, and adaptation to hypoxia. Clinical studies have yet to establish a causal link between altered lipid metabolism, abnormalities in tumor vascular architecture or perfusion, reduced intratumoral platinum exposure, and diminished OXA efficacy. Future studies in patients receiving FOLFOX or CAPOX, with or without bevacizumab, should simultaneously assess COX-2/PGE2, VEGF, HIF-1α, microvessel density, pericyte coverage, vasculogenic mimicry, and functional perfusion, while also quantifying intratumoral platinum content and platinum–DNA adducts. Only if lipid metabolic alterations are shown to modify tumor exposure to OXA and the subsequent treatment response can COX-2/PGE2 or other lipid metabolism related vascular markers be considered reliable predictors of therapeutic efficacy and targets for combination treatment.

## 8 Lipid metabolic regulation of autophagy in OXA resistance

Autophagy is a homeostatic process through which cells use lysosomes to degrade damaged proteins, organelles, and selected metabolic substrates. DNA damage, mitochondrial dysfunction, endoplasmic reticulum stress, and ROS accumulation induced by OXA can activate autophagy in CRC cells. At moderate levels, autophagy removes damaged mitochondria and oxidized proteins while maintaining energy and redox homeostasis, and therefore often serves as a cytoprotective response. However, excessive autophagy, impaired degradative flux, or cellular damage that exceeds the capacity for compensation may also promote cell death. The effect of autophagy on OXA efficacy therefore depends on autophagic flux and cellular context and cannot be inferred solely from increased LC3-II levels or autophagosome accumulation (56). Direct evidence from CRC models supports a role for OXA induced protective autophagy. HSF1/miR-135b-5p signaling is enhanced in OXA resistant cells. By suppressing the mitochondrial E3 ubiquitin ligase MUL1, miR-135b-5p reduces MUL1 mediated ubiquitination and degradation of ULK1, thereby stabilizing ULK1 and promoting autophagy initiation. Inhibition of miR-135b-5p or blockade of autophagy increased OXA induced cell death and enhanced OXA mediated tumor suppression in xenograft models. The study also found that serum miR-135b-5p levels were higher in patients with OXA resistant disease than in those who remained sensitive to OXA, suggesting that protective autophagy may be associated with clinical resistance (57). However, serum miRNA levels can be influenced by tumor burden and other factors; therefore, serum miR-135b-5p cannot yet serve as a standalone predictor of response to OXA.A separate study showed that TXNDC9 regulates the balance between autophagy and apoptosis in response to OXA. OXA treatment induced TXNDC9 expression in CRC cells, and elevated TXNDC9 favored the maintenance of autophagy while suppressing apoptosis. Conversely, TXNDC9 knockdown attenuated autophagic responses, increased apoptosis, and restored cellular sensitivity to OXA (58). These findings indicate that redox related proteins can influence cell fate decisions between autophagy and apoptosis.Studies of PFKFB3 also support a cytoprotective role for OXA induced autophagy. OXA increased PFKFB3 expression in a time and dose dependent manner, whereas PFKFB3 inhibition reduced ULK1 phosphorylation and autophagic flux and enhanced the inhibitory effects of OXA on CRC cell proliferation and survival (59). Because PFKFB3 primarily regulates glycolysis, this result supports metabolically sustained protective autophagy but does not directly implicate lipid metabolism.

Lipid metabolism and autophagy are interconnected through bidirectional mechanisms. Autophagosome formation first requires extensive remodeling of membrane lipids. The conjugation of LC3 to phosphatidylethanolamine is a critical step in the expansion and closure of the autophagic membrane, whereas phosphatidylinositol and its derivatives help recruit autophagy initiation complexes and membrane associated machinery (60). Conversely, autophagy can selectively degrade lipid droplets through a process known as lipophagy. Lysosomal degradation of triglycerides and cholesteryl esters stored in lipid droplets releases free fatty acids, which can subsequently enter mitochondrial FAO to generate ATP and metabolic intermediates (61). Thus, lipids not only provide the structural foundation for autophagic membranes but also serve as important substrates for autophagy mediated metabolic adaptation. Under OXA pressure, CRC cells may undergo a protective metabolic adaptation in which fatty acids are stored in lipid droplets, mobilized through lipophagy, and subsequently channeled into FAO (**Figure 3C**). Following OXA induced ROS accumulation and mitochondrial damage, CRC cells may initially sequester a proportion of free fatty acids in lipid droplets, thereby limiting lipotoxicity and membrane lipid peroxidation. As drug pressure persists, lipophagy may remobilize these lipid stores and provide substrates for FAO. In parallel, mitophagy can remove dysfunctional mitochondria and limit sustained ROS production. When lipid storage, lipophagy mediated fatty acid mobilization, FAO supported energy production, and mitochondrial quality control operate together, CRC cells may be better able to maintain redox homeostasis and evade OXA induced mitochondrial apoptosis.

Studies of LPCAT2 provide support for the lipid droplet component of this model. LPCAT2 promotes phosphatidylcholine synthesis and lipid droplet formation, and both LPCAT2 expression and basal lipid droplet content are associated with CRC cell tolerance to 5-FU and OXA. Increased lipid droplet accumulation attenuated endoplasmic reticulum stress, caspase activation, and cell death (47). However, the study did not assess the colocalization of lipid droplets with LC3 or lysosomes, nor did it demonstrate that lipid droplet derived fatty acids entered FAO through lipophagy. These findings therefore indicate that lipid droplets may provide a metabolic basis for protective adaptation, but do not constitute direct evidence that lipophagy drives OXA resistance.

Lipophagy may also exert context dependent, bidirectional effects on the response to OXA. Moderate lipophagic activity may provide a sustained supply of fatty acids to support FAO and the survival of residual cells. In contrast, excessive lipophagy could deplete the buffering capacity of lipid droplet stores, increasing cellular exposure to free fatty acids and polyunsaturated fatty acids and thereby exacerbating endoplasmic reticulum stress, lipid peroxidation, and ferroptosis. Conversely, impaired lipophagy may lead to abnormal lipid droplet accumulation and lysosomal dysfunction. Cell fate is therefore determined not by the absolute abundance of lipid droplets or individual autophagy markers, but by the dynamic balance among lipid droplet formation, lipophagic mobilization, FAO mediated utilization, and lipid peroxidation.A 2026 preclinical study showed that knockout of the lipid metabolizing enzyme PLA2G16 increased the sensitivity of CRC cells and xenograft tumors to OXA. PLA2G16 deficiency also altered the composition of triglycerides, phosphatidylcholine, and phosphatidylethanolamine, together with changes in LC3B-II and p62 levels and autophagosome abundance (62). Although this study provides a relatively direct link between lipid remodeling, autophagic status, and the response to OXA, the concurrent increases in LC3B-II and p62 may reflect impaired autophagic flux rather than a simple increase in autophagic activity. Furthermore, the study did not demonstrate that fatty acids stored in lipid droplets were mobilized through lipophagy and subsequently channeled into FAO. PLA2G16 may therefore represent a candidate molecular link between lipid metabolism, autophagy regulation, and OXA sensitivity, although whether its effects on the OXA response depend on lipophagy remains to be established.

Future studies should dynamically assess autophagic flux in OXA sensitive, residual cell, and stably resistant models rather than relying on LC3-II measurements alone. LC3-II and p62 should be evaluated in combination with lysosomal inhibition assays, alongside the colocalization of lipid droplets with LC3 and LAMP1, lipid droplet turnover, free fatty acid release, FAO flux, mitochondrial function, and lipid ROS.Only if blocking lipophagy is shown to reduce FAO, disrupt cellular energy or redox homeostasis, and restore sensitivity to OXA can the coordinated process of lipid droplet storage, lipophagic mobilization, and FAO mediated fatty acid utilization be considered a causal mechanism of OXA resistance in CRC rather than a set of changes that merely accompanies protective autophagy and lipid droplet accumulation.

## 9 Conclusion

Lipid metabolic reprogramming influences multiple components of OXA resistance, including DNA damage repair, redox homeostasis, ferroptosis, immunogenic cell death, and residual tumor-cell survival. Available studies have identified several potentially actionable processes, particularly lipid-droplet biogenesis, fatty acid oxidation (FAO), phospholipid remodeling, and antioxidant defense. However, most evidence is derived from cell culture and animal models, whereas clinical validation remains limited. Moreover, associations between individual lipid metabolic markers and patient prognosis do not establish their causal involvement in OXA resistance. Further studies are therefore required to determine whether these metabolic features can predict treatment response and whether their functional inhibition can restore OXA sensitivity. The evidence-tier classification of the lipid metabolic factors and pathways discussed in this review is summarized in **Table 2**.

**Table 2:** Evidence-tier classification of lipid metabolic factors associated with oxaliplatin resistance in colorectal cancer.

Evidence tier	Factors or pathways
Tier I	CPT1B/CPT2, CPT2 downregulation, ACSL4, KIF20A/NUAK1–NRF2–GPX4, *F. nucleatum*–E-cadherin/β-catenin–GPX4, exosomal MTTP–PRAP1–ZEB1, HSF1/miR-135b-5p–MUL1–ULK1, PLA2G16
Tier II	GPAT3, LPIN1/DGAT2, ELF1–ACSL5–lipid-droplet, LPCAT2, COX-2–PGE2–VEGF.
Tier III	p53–ABCA1–SREBP2, CPT1A, LPCAT3, SLC7A11/SLC3A2–GSH–GPX4, FAO–ACLY–acetyl-CoA–p300/H3K27ac–Nanog, IL-33–mTOR/PPARγ–DGAT1/2

Tier I indicates direct functional evidence from OXA-treated CRC or gastrointestinal cancer models, supported by genetic or pharmacological manipulation demonstrating an altered OXA response. Tier II indicates supportive evidence from CRC chemotherapy models or OXA-containing combination regimens, but without OXA-monotherapy-specific causality or complete in vivo validation. Tier III indicates contextual mechanistic evidence derived from non-OXA or non-CRC models, for which the relevance to OXA resistance remains inferential. None of the listed factors has yet been prospectively validated as a clinical predictor of OXA resistance.

Future research should move beyond single-marker measurements and test whether integrated lipid metabolic profiles improve the prediction of OXA response beyond conventional clinicopathological and genomic variables. Building on the framework proposed by Liu et al. (63), explainable AI models could integrate longitudinal lipidomic and transcriptomic data with lipid-droplet burden, circulating tumor DNA-defined molecular residual disease, and treatment outcomes. The candidate features should specifically represent the mechanisms discussed in this review, including lipid-droplet biogenesis mediated by GPAT3, LPCAT2, and LPIN1/DGAT2; FAO mediated by CPT1/CPT2; and the ferroptosis balance determined by ACSL4/LPCAT3 and the SLC7A11–GSH–GPX4 axis. AI should primarily be used to identify reproducible combinations of these features and prioritize candidate dependencies for experimental validation, rather than to infer causality from observational data alone. Consistent with the concerns raised by Truhn et al. (64), model interpretability, independent validation, data security, and continued oversight by researchers and clinicians remain essential.

Patient-derived organoids provide a suitable platform for testing whether AI-prioritized lipid metabolic features represent causal resistance mechanisms (65). Organoids established from pretreatment and recurrent tumors could be compared with residual-like and stably resistant populations generated through controlled OXA-monotherapy exposure. Genetic or pharmacological perturbation of the prioritized pathways should be followed by measurements of OXA sensitivity, regrowth after drug withdrawal, lipid-droplet turnover, FAO flux, PUFA-containing phospholipids, lipid ROS, and ferroptosis rescue. These experiments would help distinguish functional resistance drivers from correlative biomarkers. Co-culture with adipocytes, fibroblasts, or immune cells and organ-on-chip systems could further test whether exogenous lipid supply and microenvironmental interactions modify these dependencies.Because residual tumor cells may adopt drug-tolerant persister, dormant, or senescence-like states, these phenotypes should not be defined by a single marker. As emphasized by Martin et al. (66), cellular senescence is temporally and contextually heterogeneous. Longitudinal profiling and lineage tracing should therefore be incorporated into organoid studies to determine whether lipid metabolic interventions eliminate residual cells or merely induce a reversible growth-arrested state.

Ultimately, combining AI-based identification of lipid metabolic signatures, causal validation in organoid models, and prospective clinical assessment may identify patients most likely to benefit from metabolism-directed treatment. This strategy could also provide a mechanistic basis for combining OXA with carefully selected inhibitors of lipid-droplet formation or FAO, ferroptosis-inducing strategies, autophagy modulators, or immunotherapy.
